# Efficacy of Low-Level Laser Therapy for Oral Mucositis in Hematologic Patients Undergoing Transplantation: A Single-Arm Prospective Study

**DOI:** 10.3390/jpm13111603

**Published:** 2023-11-13

**Authors:** Hiromi Nishi, Susumu Horikoshi, Kouji Ohta, Tetsumi Yoshida, Noriyasu Fukushima, Kyoko Oshita, Syuichi Munenaga, Taro Edahiro, Hiroshi Ureshino, Hideo Shigeishi, Yukio Yoshioka, Masaru Konishi, Noriaki Ide, Yuma Ogawa, Rikou Marukawa, Tomoaki Shintani, Natumi Ino, Mikihito Kajiya, Naoya Kakimoto, Hiroki Ohge, Tatsuo Ichinohe, Hiroyuki Kawaguchi

**Affiliations:** 1 Department of General Dentistry, Hiroshima University Hospital, 1-2-3, Kasumi, Minami-Ku, Hiroshima 734-8551, Japan; horiko@hiroshima-u.ac.jp (S.H.); syu-munenaga@hiroshima-u.ac.jp (S.M.); noriide@hiroshima-u.ac.jp (N.I.); hkawarp@hiroshima-u.ac.jp (H.K.); 2 Department of Public Oral Health, Program of Oral Health Sciences, Graduate School of Biomedical and Health Sciences, Hiroshima University, Hiroshima 734-8551, Japan; otkouji@hiroshima-u.ac.jp (K.O.); shige@hiroshima-u.ac.jp (H.S.); 3 Department of Hematology and Oncology, Research Institute for Radiation Biology and Medicine, Hiroshima University, Hiroshima 734-8551, Japan; yoshidat@hiroshima-u.ac.jp (T.Y.); tedahiro@hiroshima-u.ac.jp (T.E.); hureshin@hiroshima-u.ac.jp (H.U.); nohe@hiroshima-u.ac.jp (T.I.); 4 Department of Internal Medicine, Karatsu Red Cross Hospital, Karatsu 847-8588, Japan; noriyasu-fukushima@karatsu.jrc.or.jp; 5 Department of Anesthesiology, Hiroshima General Hospital, Hiroshima 734-8551, Japan; oshitak@hiroshima-u.ac.jp; 6 Department of Oral Oncology, Graduate School of Biomedical and Health Sciences, Hiroshima University, Hiroshima 734-8551, Japan; yyosioka@hiroshima-u.ac.jp; 7 Department of Oral and Maxillofacial Radiology, Hiroshima University Hospital, Hiroshima 734-8551, Japan; mkonishi@hiroshima-u.ac.jp; 8 Department of Program of Dentistry, School of Dentistry, Hiroshima University, Hiroshima 734-8551, Japan; b206290@hiroshima-u.ac.jp (Y.O.); b204686@hiroshima-u.ac.jp (R.M.); 9 Center of Oral Clinical Examination, Hiroshima University Hospital, Hiroshima 734-8551, Japan; tshintan@hiroshima-u.ac.jp (T.S.); mkajiya@hiroshima-u.ac.jp (M.K.); 10 Department of Clinical Practice and Support, Hiroshima University Hospital, Hiroshima 734-8551, Japan; 11 Department of Oral and Maxillofacial Radiology, Graduate School of Biomedical and Health Sciences, Hiroshima University, Hiroshima 734-8553, Japan; kakimoto-n@hiroshima-u.ac.jp; 12 Department of Infectious Diseases, Hiroshima University Hospital, Hiroshima 734-8551, Japan; ohge@hiroshima-u.ac.jp

**Keywords:** oral mucositis, hematopoietic stem cell transplantation, low-level light therapy, quality of life, Japan

## Abstract

Oral mucositis significantly affects the quality of life in hematologic cancer patients undergoing hematopoietic stem cell transplantation. Despite global evidence supporting the efficacy of low-level laser therapy (LLLT) for mucositis prevention, its clinical adoption in Japan is limited. This study aimed to fill this gap by evaluating the safety and efficacy of LLLT in a Japanese patient population. In a single-group, non-blinded, exploratory trial, we compared 21 LLLT-treated patients against a historical control of 96 patients. The primary endpoint was the incidence of Grade ≥ 2 mucositis, based on NCI-CTCAE ver. 4.0. The LLLT group showed a significantly lower incidence of Grade ≥ 2 mucositis (23.8%) compared to the control group (64.6%) (*p* = 0.0006). Furthermore, Grade ≥ 2 mucositis correlated with increased oral dryness and longer hospital stays. Our study confirms the efficacy of LLLT in reducing the onset of severe oral mucositis among Japanese hematologic cancer patients, advocating for its clinical introduction as a preventive measure in Japan.

## 1. Introduction

Oral mucositis is recognized as a side effect in patients who receive chemotherapy or radiotherapy treatments. Pain caused by oral mucositis, as well as an inability to eat and the development of local infection can markedly diminish patient quality of life, often resulting in prolonged hospitalization and the interruption of cancer treatment. Furthermore, compromised oral intake due to mucositis leads to malnutrition and weight loss, which are associated with cancer prognosis and survival [1,2]. In particular, oral mucositis has been found to develop in 76–89% of patients with hematologic malignancy who undergo high-dose chemotherapy as a pre-treatment procedure before transplantation, while its development has been reported in 100% of patients who underwent total body irradiation [3]. Moreover, another study indicated that 65% of patients in Japan undergoing autologous or allogeneic hematopoietic stem cell transplantation develop oral mucositis Grade ≥ 2, as classified by NCI-CTCAE, ver. 4.0 [4].

The establishment of fundamental treatments for oral mucositis resulting from cancer therapy remains elusive, with symptomatic methods aimed at symptom relief being the predominant approach. With such palliative strategies, oral hygiene practices are emphasized to mitigate the risk of secondary infections from oral bacteria associated with oral mucositis and encompass an intervention that targets potential infectious sources in the oral cavity, such as dental caries and periodontal disease, as well as measures to sustain optimal oral bacterial levels. For patients with oral mucositis presenting with pain, anti-inflammatory analgesic treatment is advocated for, along with supplementation with supportive therapy, employing agents such as Azunol^®^ ointment, honeybee azulene gargle, and xylocaine ointment [5]. Cryotherapy has been reported to offer efficacy in a subset of patients, with findings showing that retaining ice chips in the mouth during treatment exploits the vasoconstrictive cooling properties, thus attenuating the manifestation of oral mucositis [6]. However, these approaches have limitations, and there is demand for new effective treatments.

In recent years, several reports have been presented that show the efficacy of low-level laser therapy (LLLT) for preventing chemotherapy-induced oral mucositis, including findings demonstrating a reduction in inflammation and promotion of tissue repair [7,8,9]. International studies have confirmed the effectiveness of LLLT for reducing the incidence and severity of oral mucositis, and it is recommended in the clinical practice guidelines presented by the Multinational Association of Supportive Care in Cancer (MASCC) [10,11]. However, this treatment is not standard in Japanese medical institutions, and no reports regarding the efficacy and safety of LLLT for preventing oral mucositis in Japan have been published.

Additionally, recent studies have investigated factors affecting the frequency and severity of oral mucositis following hematopoietic stem cell transplantation. These factors include the type of conditioning chemotherapy, the use of methotrexate (MTX) for GVHD prophylaxis, prior history of craniospinal (CSI) radiation, and the patient’s age. Studies have shown that patients who received myeloablative conditioning regimens, those who were administered prophylactic MTX, and those with a history of prior CSI radiation were more likely to develop oral mucositis [12]. This information highlights the importance of systemic factors in developing oral mucositis and provides valuable insights into potential risk factors.

With this background in mind, the present study was conducted to assess the efficacy and safety of LLLT for preventing oral mucositis by evaluating the grade of oral mucositis in patients in Japan who underwent stem cell transplantation for hematologic cancer and received LLLT. This exploratory study was conducted as a single-arm, non-blinded trial with a historical control. The primary endpoint was the incidence of oral mucositis Grade ≥ 2, based on NCI-CTCAE, ver. 4.0.

## 2. Materials and Methods

### 2.1. Study Design

In this prospective, single-center, single-arm, non-blinded, non-randomized study, the efficacy of LLLT for mitigating chemotherapy-induced oral mucositis and alleviating related symptoms was evaluated in patients undergoing autologous or allogeneic stem cell transplantation in Japan. The effects of LLLT were compared to a historical control group of patients who did not receive that therapy, previously reported by Ohbayashi et al. [4]. This trial was conducted at Hiroshima University Hospital from December 2022 to September 2023. Eligible participants met the following inclusion criteria: they were aged 18 or older at the time of consent, were diagnosed with hematologic malignancies and scheduled for either allogeneic or autologous stem cell transplantation (not limited to initial treatment), had no oral mucositis from other chemotherapies in pre-observation, and provided written consent. Exclusion criteria included the presence of oral mucositis from physical irritants such as dental caries or ill-fitting dentures, a history of prior radiation therapy including the oral area, or being deemed unsuitable for the study by the lead or co-investigators.

### 2.2. Criteria for Treatment Discontinuation

Criteria for discontinuing LLLT treatment for the prevention of oral mucositis in the present subjects were as follows. (1) Withdrawal of consent for study participation by the patient. (2) Deterioration of the primary disease, complications, or adverse events leading to a judgment that LLLT is not advisable. (3) Determination that fewer than three of the total ten therapy sessions will be administered. There were no instances where laser therapy for any of the present subjects was discontinued after initiation.

### 2.3. Prophylactic LLLT for Oral Mucositis

Two dentists administered LLLT to the oral cavity of the present subjects for 10 consecutive days, beginning from the start of the pre-treatment, i.e., the intensive chemotherapy regimen administered to patients with hematologic malignancy before undergoing transplantation. Each low-level laser treatment (wavelength 650 nm, output 40 mW, energy density 2 J/cm^2^, and 2 s per point) targeted the upper and lower lips (both vermillion and inner surfaces), buccal mucosa on both sides, soft palate, dorsal surface of the tongue, lateral borders of the tongue, and floor of the mouth. Utilizing the guiding light of the laser handpiece, the laser was uniformly applied to minimize untreated areas. Each treatment session lasted approximately 10 min.

Before the initiation of pre-treatment, the oral cavity was examined to identify infectious lesions. When those were identified, appropriate intervention was administered, including tooth extraction, periodontal treatment, infected root canal treatment, and caries treatment. Concurrently, oral hygiene instructions were provided to complement those therapeutic measures. From the start of pre-treatment, oral hygiene management was conducted twice a week using toothbrushes and tongue brushes. To prevent oral dryness, honeybee azulene gargle was used from the initiation of pre-treatment. In patients with oral mucositis with an NCI-CTCAE, ver. 4.0, Grade of 1 or higher, anti-inflammatory analgesics, azunol ointment, xylocaine ointment, and local management with Episil^®^ Oral Liquid, a hydrogel wound covering/protective agent, were employed based on the symptoms. Additionally, oral cryotherapy was performed by holding ice chips in the mouth 30 min before the administration of melphalan and busulfan, for leveraging the vasoconstrictive effects of cooling.

### 2.4. Oral Assessment and Measurements

The oral conditions of patients enrolled in the present study were evaluated every three days by two dentists, starting from the initiation of pre-treatment. Oral mucositis was assessed based on NCI-CTCAE, ver. 4.0. Specifically, Grade 1 was defined as asymptomatic or mild symptoms with no intervention indicated; Grade 2 as moderate pain or ulcer that does not interfere with oral intake, with a modified diet indicated; and Grade 3 as severe pain, interfering with oral intake.

Oral bacteria were quantified using a bacterial counter (Panasonic Healthcare Holdings Co., Ltd., Tokyo, Japan) based on electrical impedance. Samples swabbed from the center of the tongue using a sterile cotton swab were measured in accordance with the manufacturer’s manual, with values below 10 million considered to be normal for oral bacterial count. Xerostomia was assessed using a Mucus Oral Hygrometer (Life Co., Ltd., Tokyo, Japan), with oral dryness defined when the average value of three measurements taken at the center of the tongue was 27 or less, according to the manufacturer’s guidelines. These measurements were conducted as previously described [13,14]. Initial measurements were obtained at the time of patient registration, followed by assessments on days 1, 6, and 10 after pre-treatment initiation, every five days. After the completion of laser irradiation, measurements were again performed on days 16, 22, and 28.

Periodontal status was evaluated at the time of patient registration using a Williams periodontal probe (HuFriedy, Chicago, IL, USA) at six sites of each fully erupted tooth (buccal anterior, lingual anterior, buccal, lingual, buccal posterior, and lingual posterior). Subsequently, periodontal epithelial surface area (PESA), representing the total surface area of the periodontal pockets, and periodontal inflamed surface area (PISA), the total surface area of periodontal pockets with inflammation due to periodontitis, were calculated using methods noted in our previous research and also those reported by Nesse et al. [15,16].

### 2.5. Endpoints

The primary efficacy endpoint was the incidence rate of oral mucositis determined to be Grade ≥ 2, based on NCI-CTCAE, ver. 4.0. The observation period was from the start of the pre-treatment period up to 28 days. Within this timeframe, the proportion of patients who developed Grade ≥ 2 oral mucositis at least once was compared with a historical control [4]. As secondary endpoints, oral bacterial count and xerostomia at the evaluation points were assessed among patients with and without Grade ≥ 2 oral mucositis. Additionally, the incidence rate of fever above 37.5 °C, frequency of missed meals (evaluated based on three meals a day), and duration until discharge during the 28-day period following the initiation of pre-treatment were compared.

### 2.6. Equipment Used and Adverse Events

For this study, a Diode Laser Sheep 810 low-power laser device was utilized (Unitac Co., Ltd., Onomichi City, Japan, medical device approval number: 22700BZX00370000). The operating principle of this device when used at low power is based on photobiological effects. Specifically, fibroblasts exposed to low-power laser irradiation have been reported to exhibit enhanced collagen synthesis due to increased ATP production in mitochondria possessing photoreceptors [17]. The photobiological action occurs at a level that does not produce thermal effects; hence, there have been no reports of thermal burns. Its safety has been established for all applications except for reproductive organs. However, there is a risk of blindness if the laser light enters the eye, so both the operator and the patient wear protective glasses during irradiation [18]. No adverse events related to the use of low-power lasers have been reported. On the other hand, when such a device is used at high power, the laser light is absorbed by biological tissues and then converted into heat. This thermal energy is used for incising soft biological tissues, hemostasis, and blood coagulation [19]. For the present study, a laser device that has received medical device approval was employed and used at a lower power than described for its medical indications.

### 2.7. Statistical Analysis

All analyses were conducted using the JMP Pro 15 statistical software package (SAS Institute Inc., Cary, NC, USA). Continuous variables are presented as mean ± standard deviation. Univariate analyses were performed to evaluate differences among patients with and without oral mucositis of Grade ≥ 2 regarding basic characteristics, oral evaluation metrics (periodontal assessment, oral bacterial count, and oral dryness), frequency of fever and missed meals, and days to discharge. As appropriate, comparisons between the groups were made using Student’s *t*-test, a Mann–Whitney U test, Fisher’s exact test, or a chi-squared test. A *p* value less than 0.05 was considered to indicate statistical significance.

### 2.8. Sample Size

In Japan, the incidence rate of Grade ≥ 2 oral mucositis without the use of a low-level laser has been reported to be 65% [4]; thus, the threshold used in the present study was 65%. On the other hand, the incidence rate of oral mucositis with the use of a low-level laser was set at an expected value of 35%, with reference to rates observed in international studies. Considering a significance level of 5% (two-sided) and a power of 80%, the required sample size was calculated to be 20 cases using a normal approximation for the binomial test. To account for potential data loss and unforeseen events, the present sample size was slightly increased and included 21 patients.

### 2.9. Data Management

All cases were documented using the REDcap data collection system (Electronic Data Capture, Hiroshima University, Hiroshima, Japan). The information obtained was aggregated without including identifiable personal data, thus ensuring data reliability and protecting individual privacy.

## 3. Results

### 3.1. Patient Characteristics

The clinical characteristics of the patients are presented in Table 1. There were 13 males (61.9%) and eight females (38.1%), with an average age of 52.6 ± 12.4 years. Regarding the type of disease, myelogenous leukemia was most common and found in eight patients, of which seven had acute myeloid leukemia and one had myelomonocytic leukemia. There were six patients with lymphocytic leukemia/lymphoma, including four with malignant lymphoma, one with acute lymphoblastic leukemia, and one with adult T-cell leukemia. Additionally, multiple myeloma was noted in three patients, virus-related disorders in two, chronic granulomatous disease in one, and amyloidosis in one. Regarding the type of hematopoietic stem cell transplantation (HSCT), 10 patients (47.6%) underwent autologous and 11 (52.4%) allogeneic HSCT. In allogeneic transplantation, fludarabine 30 mg/m^2^/d × 4 days and melphalan 140 mg/m^2^ × 1 day (FluMel140) is a commonly used RIC regimen [20]. In this study, nine patients (42.9%) underwent Reduced-Intensity Conditioning (RIC) with regimens such as FluMel140, as well as other drugs using melphalan at doses of 140 mg/m^2^ or lower. On the other hand, 12 patients (57.1%) were treated under the Myeloablative Conditioning (MAC) regimen, which employed more intensive regimens than RIC. Specifically, in the MAC regimen, combinations such as fludarabine 30 mg/m^2^/d × 4 days plus busulfan 3.2 mg/kg/d × 4 days (FluBu4) or FluBu4 and Mel 140 mg/m^2^ × 1 day (FluBu4-Mel140) were utilized [21]. In autologous transplantation, other MAC regimens like melphalan 100 mg/m^2^/d × 2 days (Mel200) and the MEAM protocol, consisting of the combination of ranimustine (MCNU) 300 mg/m^2^/d, etoposide 200 mg/m^2^/d × 4 days, cytarabine 400 mg/m^2^/d, and melphalan 140 mg/m^2^/d, were also used. Concerning total body irradiation (TBI), fourteen patients (66.7%) did not undergo TBI, while seven (33.3%) received 2 Gy-TBI.

### 3.2. Efficacy of Low-Level Laser Therapy for Oral Mucositis

**Figure 1:**
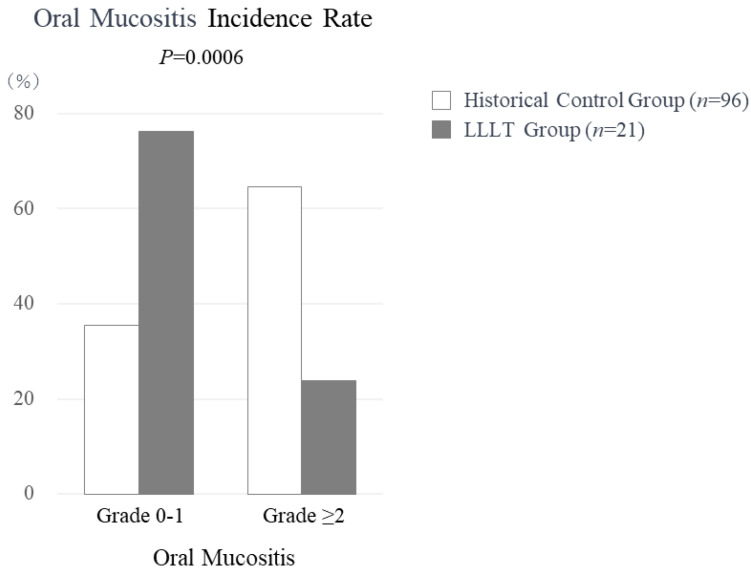
The incidence rate of oral mucositis.

Findings showing the effects of LLLT on oral mucositis are presented in Figure 1. Among the twenty-one treated patients, two developed Grade 1 oral mucositis, five developed Grade 2, and none developed Grade 3. The incidence rate of oral mucositis of Grade 2 or above was 23.8% (5 of 21). In contrast, in the historical control group that did not receive LLLT (*n* = 96), Grade 1 oral mucositis was noted in 8, Grade 2 in 29, and Grade 3 in 33, for an incidence rate of Grade ≥ 2 oral mucositis of 64.6% (62 of 96). There was a significant difference for the incidence of oral mucositis Grade ≥ 2 between the present study group that received LLLT and the historical control group that did not (*p* = 0.0006).

### 3.3. Low-Level Laser Therapy Safety Profile

None of the present patients exhibited complications associated with laser treatment. Furthermore, there were no instances where treatment had to be discontinued. Based on these findings, it was concluded that low-level laser therapy is safe for Japanese patients with hematologic malignancies.

### 3.4. Comparison of Patient Characteristics Based on Oral Mucositis Severity

The clinical characteristics of the patients based on mucositis severity are shown in Table 2. There were no significant differences for age, gender, and BMI between the groups with Grade 0–1 and those with Grade ≥ 2. Evaluative parameters for periodontal disease, such as PESA (periodontal epithelial surface area) and PISA (periodontal inflamed surface area) values, oral bacterial count, and oral dryness, which were assessed at the time of patient registration, also showed no significant variances between the groups. As for treatment-related characteristics, in the Grade ≥ 2 group, the incidence of allogeneic HSCT was 80.0% (*n* = 4) and that of autologous HSCT was 20.0% (*n* = 1), with no significant disparity observed between them. In terms of donor source, autologous peripheral stem cells were observed in one (20.0%), related bone marrow in one (20.0%), and unrelated bone marrow in three (60.0%) patients in the Grade ≥ 2 group. A significant difference in regard to donor source was noted between the groups (*p* < 0.05). Regarding post-treatment outcomes in the Grade ≥ 2 group, the number of days with fever after pre-treatment was 6.8 ± 4.3 days and the number of instances of fasting was 11.8, with counting based on three meals a day as the standard. No significant differences were found for these parameters between the groups. On the other hand, the duration from pre-treatment to discharge for patients in the Grade ≥ 2 group was 68.8 ± 24.4 days, a significant difference (*p* < 0.05).

### 3.5. Temporal Changes in Oral Bacterial Count following Pre-Treatment Initiation

**Figure 2:**
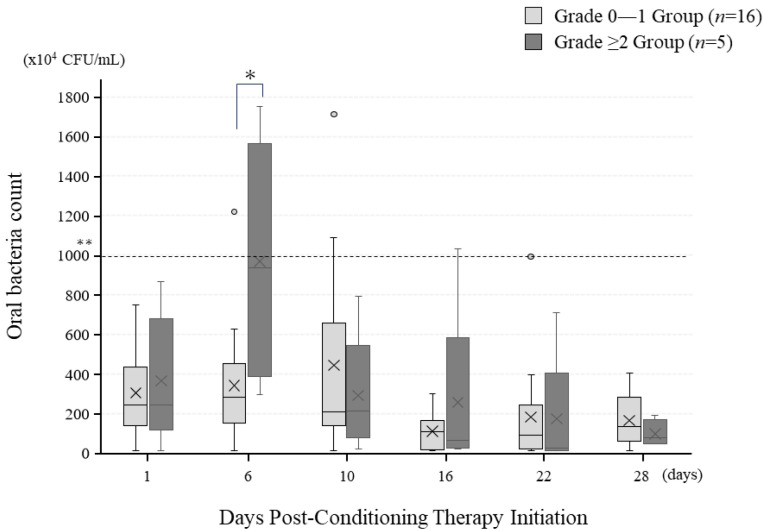
Temporal changes in oral bacterial count. The “×” mark represents the average value at each time point. * *p* < 0.05, ** Value ≤ 1000 × 10^4^ is normal; >1000 × 10^4^ indicates a high oral bacteria count.

Changes in oral bacterial count for the oral mucositis Grade 0–1 and Grade ≥ 2 groups are illustrated in Figure 2. In both groups, bacterial count remained within the normal range of less than 1000 at all examined time points, though a significant difference was observed between the groups at six days after pre-treatment initiation (*p* = 0.01). At the other time points, no significant differences between the groups were noted.

### 3.6. Temporal Changes in Oral Dryness following Pre-Treatment Initiation

**Figure 3:**
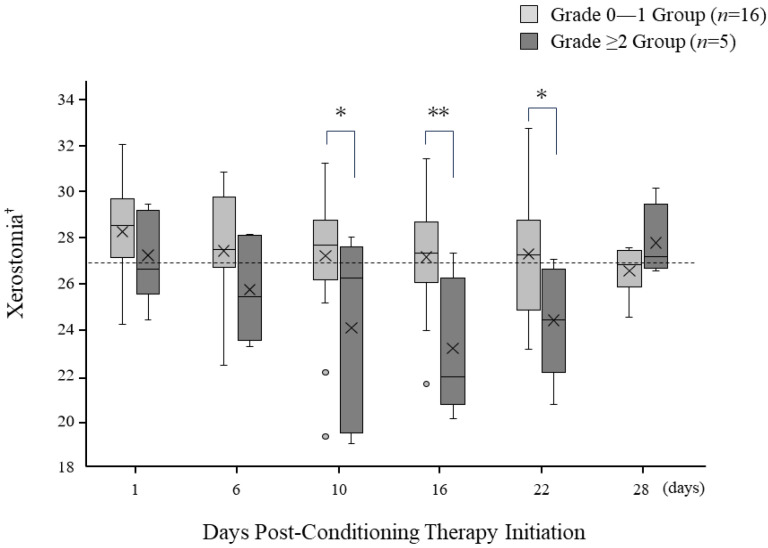
Temporal changes in oral dryness. The “×” mark represents the average value at each time point. * *p* < 0.05, ** *p* < 0.01. ^†^ Threshold for oral dryness is a value > 27. Values are relative measurements; there are no specific units.

Changes in oral dryness for the Grade 0–1 and Grade ≥ 2 groups are presented in Figure 3. Xerostomia level was measured using a specialized device, with the threshold defined as a value below 27, though the values obtained were relative. On the day of pre-treatment initiation, patients in both groups demonstrated a dryness level within the normal range. However, as the days progressed, the Grade ≥ 2 group showed a marked increase in oral dryness, falling significantly below the threshold on day 6 after initiation. This increase persisted until post-initiation day 16, after which a recovery trend was noted and there was a return to the normal range by day 28. In contrast, patients in the Grade 0–1 group consistently maintained a dryness level near the standard threshold throughout the study period. Significant differences between the two groups were observed on days 10 (*p* = 0.04), 16 (*p* = 0.006), and 22 (*p* = 0.03).

## 4. Discussion

The present results suggest that LLLT is effective to prevent oral mucositis in patients undergoing treatments in Japan. In a study by Ohbayashi et al. [4], used as a historical control in the present examinations, 62 out of 96 patients (64.6%) developed oral mucositis Grade ≥ 2. In contrast, among the 21 patients in the present study who received LLLT, the Grade 2 oral mucositis incidence rate was limited to 23.8% and no cases of Grade 3 were observed. As compared with the results of Ohbayashi et al. [4], the incidence rate was notably lower, strongly indicating the efficacy of LLLT.

Based on the MASCC/ISOO guidelines, LLLT was administered for 10 days in the present study cohort, utilizing a wavelength of 650 nm, an output of 40 mW, and a radiation energy density of 2 J/cm^2^ [10,22]. This regimen has been confirmed to effectively prevent oral mucositis in a Japanese population. However, some cases showed oral mucositis development after the 10-day irradiation period, suggesting that further evaluation of the preventive effects and safety of extended irradiation beyond 10 days is required. The preventive effects against oral mucositis are reported to vary based on energy density and irradiation duration. Antunes HS et al. indicated that the effectiveness of LLLT increases as energy density and irradiation time increase [23]. Nevertheless, a unified standard for the optimal conditions of LLLT is needed, especially regarding the wavelength and output parameters. Current treatment protocols for LLLT are primarily based on individual research findings and experiences. It is considered that additional clinical trials and the establishment of more unified guidelines are required.

Oral bacteria are associated with the progression of oral mucositis occurring during cancer treatment. The proliferation of bacteria and subsequent colony formation exacerbate tissue damage associated with mucositis, while infiltration by mononuclear cells further activates the production and release of inflammatory cytokines, promoting the progression of oral mucositis [24]. Additionally, there is a direct relationship between the exacerbation of xerostomia and the severity of oral mucositis. Jones demonstrated that for every 5% increase in oral dryness, the severity of oral mucositis is increased by a grade of 0.5 [25]. In the present study, there was no significant difference between the two groups regarding oral bacterial count or xerostomia on the day pretreatment started. However, in the Grade ≥ 2 group, there was a significant increase in oral bacterial count at six days following post-pretreatment initiation. Furthermore, between days 10 and 22, there was a significant difference in exacerbation of oral dryness between the groups. These findings suggest that more frequent oral care before and after transplantation, aimed at reducing oral bacterial count and alleviating oral dryness, may potentially reduce the severity of oral mucositis. Alongside these considerations, it is worth exploring alternative or complementary treatments. For instance, ozone therapy has been studied for its antibacterial and anti-inflammatory effects in periodontal disease, showing promise as a comparable treatment to laser therapy [26,27]. Given that our study focuses on the efficacy of LLLT, incorporating ozone therapy in future research could offer a multifaceted approach to managing oral mucositis.

It has been reported that the incidence of oral mucositis as well as its severity vary depending on the type of transplant (autologous transplant; allogeneic transplant with MAC: Myeloablative Conditioning; RIC: Reduced-Intensity Conditioning) [28]. In particular, the MAC regimen, which involves high-dose melphalan or busulfan treatments, is used as a conditioning regimen before bone marrow transplantation. Because of its myeloablative characteristics, it is considered to increase the risk of oral mucositis development [29]. However, there were no significant differences observed in the present study for the onset of oral mucositis between MAC and RIC regimens. A decrease in severe oral mucositis caused by LLLT may explain the lack of differences among types of transplants, different than noted in previous reports.

Regarding donor source, a significant difference was observed between the Grade 0–1 and Grade ≥ 2 oral mucositis groups. Of the patients who underwent autologous peripheral blood stem cell transplantation, 56.3% and 20.0% developed Grade 0–1 and Grade ≥ 2, respectively, while those values for patients who underwent unrelated bone marrow transplantation were 12.5% and 60.0%, respectively. Previous reports have indicated that donor source has an influence on the onset and severity of oral mucositis, which was also noted in the present findings. There is a risk of graft-versus-host disease associated with unrelated bone marrow, umbilical cord blood, or umbilical cord blood transplantation, with differences in immune responses proceeding to severe oral mucositis [30,31]. Therefore, it may be necessary to improve the current protocol in regard to frequency, irradiation time, and intensity for patients undergoing unrelated bone marrow transplantation.

This study has some limitations. This was an exploratory examination that used findings obtained in a single-arm, non-blinded trial, along with a historical control, based on procedures conducted at a single institution. Thus, the effects of LLLT noted, as compared to previous reports, may have been influenced by differences in cancer treatment. In the future, it will be necessary to investigate the effects of LLLT on preventing oral mucositis through the use of randomized controlled trials and multi-institutional joint research studies.

The MASCC has published clinical guidelines for preventing oral mucositis during cancer treatment and recommends LLLT as a possible intervention method [32]. However, the application of LLLT has yet to be adopted in Japan because of a lack of domestic clinical trial data and no establishment of national guidelines regarding supportive cancer care. Therefore, the present study examined the effects of LLLT on the development of oral mucositis in hematologic cancer patients in Japan during chemotherapy by comparing a single group of LLLT patients with a historical control group. The findings suggest that LLLT is effective for preventing the development of oral mucositis, as indicated in previous reports and noted in guidelines used in other countries. In addition, they support the clinical application of LLLT as a new preventive method for oral mucositis during cancer treatment for patients in Japan, and provide a valuable reference for future treatment policies and new guidelines.

## 5. Conclusions

Based on the findings of this study, it is imperative to undertake the following measures to proactively promote the adoption of LLLT, not only in Japan but also on a global scale. These measures include the development of comprehensive national guidelines, initiation of randomized comparative trials, and the establishment of collaborative research networks among international healthcare institutions. Such proactive measures are expected to enhance the quality of life for patients and significantly contribute to the advancement of cancer care worldwide.

## Figures and Tables

**Table 1:** Baseline clinical characteristics in this study (*n* = 21).

Clinical Parameter	*n* = 21
Age (mean ± SD)	52.6 ± 12.4
Gender (% male)	13 (61.9)
BMI (mean ± SD)	23.5 ± 3.1
Disease, n (%)	
-Myeloid Leukemias	8 (38.1)
-Lymphoid Leukemias/Lymphomas	6 (28.6)
-Multiple Myeloma	3 (14.3)
-Virus-Related Disorders	2 (9.5)
-Other Disorders	2 (9.5)
Type of Hematopoietic Stem Cell Transplantation (HSCT), n (%)	
-Autologous-HSCT	10 (47.6)
-Allogeneic-HSCT	11 (52.4)
Donor source, n (%)	
-Autologous Peripheral Blood Stem Cell	10 (47.6)
-Related Bone Marrow	1 (4.8)
-Unrelated Bone Marrow	5 (23.8)
-Unrelated Cord Blood	5 (23.8)
Conditioning regimen, n (%)	
-Reduced-Intensity Conditioning (RIC)	9 (42.9)
-Myeloablative Conditioning (MAC)	12 (57.1)
Total Body Irradiation (TBI), n (%)	
-Non-TBI	14 (66.7)
-2Gy-TBI	7 (33.3)

**Table 2:** Comparison of various factors between Grade 0–1 and Grade ≥ 2 oral mucositis groups.

Parameter	Grade 0–1 Oral Mucositis (*n* = 16)	Grade ≥ 2 Oral Mucositis (*n* = 5)	*p* Value
Age, years	55.2 ± 3.1	46.6 ± 5.6	0.19
Gender (% male)	9 (56.3)	4 (80.0)	0.32
BMI (mean ± SD)	22.8 ± 3.1	25.4 ± 2.2	0.10
PESA (mm^2^), median (IQR)	1240.8 (876.8–1353.4)	1001.3 (823.2–1145.9)	0.61
PISA (mm^2^), median (IQR)	45.8 (23.7–209.6)	24.6 (3.0–112.2)	0.31
Oral bacteria count (×10^4^ CFU/mL)	478.2 ± 336.0	827.8 ± 365.9	0.09
Xerostomia (mean ± SD)	26.5 ± 3.3	27.8 ± 2.7	0.50
Type of Hematopoietic Stem Cell Transplantation (HSCT), n (%)			0.14
-Autologous-HSCT	9 (56.3)	1 (20.0)	
-Allogeneic-HSCT	7 (43.8)	4 (80.0)	
Donor source, n (%)			<0.05
-Autologous Peripheral Stem Cell	9 (56.3)	1 (20.0)	
-Related Bone Marrow	0 (0.0)	1 (20.0)	
-Unrelated Bone Marrow	2 (12.5)	3 (60.0)	
-Unrelated Cord Blood	5 (31.3)	0 (0.0)	
Conditioning regimen, n (%)			0.88
-Reduced-Intensity Conditioning	7 (43.8)	2 (40.0)	
-Myeloablative Conditioning	9 (56.3)	3 (60.0)	
Total Body Irradiation (TBI), n (%)			0.45
-Non-TBI	10 (62.5)	4 (80.0)	
-2Gy-TBI	6 (37.5)	1 (20.0)	
Days with Fever after Pre-treatment	6.1 ± 4.2	6.8 ± 4.3	0.74
Missed Meals after Pre-treatment	6.2 ± 3.8	11.8 ± 6.8	0.48
Days from Pre-treatment to Discharge	41.5 ± 24.3	68.8 ± 24.4	<0.05

## Data Availability

The data supporting this study’s findings will be available from the corresponding author upon reasonable request.
